# Abstracts and Gleanings

**Published:** 1880-08-20

**Authors:** 


### ABSTRACTS AND GLEANINGS.

**Gelsemium.**—If the face is flushed, the eyes bright, the pupils contracted, the temperature elevated, the muscles twitching, and the tongue tremulous, give gelsemium. If the temperature is normal or below, the eyes dull, the pupils dilated, the mind cloudy, the pulse feeble, and no nervousness, do not give gelsemium.

Children while suffering from high fever or irritation from any source are especially liable to convulsions. Under such conditions gelsemium is the remedy *par excellence*, as a nerve sedative to lessen the liability to convulsions.

This drug seems to produce such a variety of symptoms shown under different circumstances and recorded by different observers, and is recommended for so many different diseases, that it has not yet been definitely classed; but it so universally allays nervous excitability from whatever cause, that it can very properly be classed, as Binz classes it, with the nervine sedatives.

Knowing the physiological action of gelsemium upon muscular fibers, in September, 1877, I administered it in a case of retention of urine from spasmodic contractions of the mouth of the bladder. The catheter could not be introduced, and chloroform was not at hand. I was then driven to try gelsemium, and, to my surprise, with the happiest results. After a few hours, before I returned with chloroform, the urine passed off naturally, and by continuing its use a few days the spasms did not return. I have since seen that others have used it, as I have since done for the same purpose, nearly always obviating the necessity of catheterization under chloroform.

In those afterpains of multiparæ, where the continued tonic pains are due to an irritability of the overwrought nervous system, rather than to a physiological process of subinvolution, gelsemium is a most excellent remedy. I give the tincture in twenty-five-drop doses every hour, and have never failed to sedate the hyperirritability of the pelvic nervous system which generally exists during subinvolution. According to my note-book, this treatment has been almost immediately successful in forty-six cases.

The pains of dysmenorrhœa and the "nagging" pains of the first stage of labor are greatly alleviated by it.

Neuralgia of the fifth nerve from temporary eccentric irritations not instances of tic douloureux, intercostal neuralgia, and myalgia are frequently cured by this agent when largely administered.

In cases of neuralgia of the trigeminus, Dr. Massini gives twenty minims of the tincture every half hour for three or four doses, and he finds that the first dose usually affords relief, and that the pain rapidly subsides after a second or third dose has been taken. He has never found it necessary to exceed sixty-minim doses, and only in one case did this quantity produce any unpleasant head symptoms. The cases in which the remedy produces most benefit are those of simple rheumatic neuralgia of the alveolar branches of the trigeminus. In these it rarely fails. It also sometimes relieves the pain remaining after the stopping of a carious tooth.

In diseases of the respiratory organs characterized by irritation, having its seat or origin in the pulmonary tissues—such, for instance, as hectic—gelsemium has acted well when all the favorite remedies for that symptomatic trouble have failed.

Gelsemium is of great service in convulsive or spasmodic cough, whooping-cough, reflex cough from irritation, hysterical cough, and in some cases of spasmodic asthma, spasmodic muscular cramps, and indeed in all troubles of a spasmodic nature that are due to nervous irritations, producing some sort of muscular contractions.

It is extolled very highly by some writers as an unfailing remedy in the early stages of acute gonorrhœa. The fluid extract is given four times a day, beginning with eight drops and increasing two drops every dose until the patient experiences the peculiar intoxication.

In 1870, Dr. E. A. Anderson made a series of experiments upon the antiperiodic properties of gelsemium, and according to him it made an excellent substitute for the cinchona barks. Dr. W. W. Murray used it subsequently with success in a large number of cases of intermittents; but I do not think its antiperiodic properties have since received confirmation, at least not by coming into general use. Its chief indication in this class of cases is to allay nervous excitement. I invariably give it in all classes of malarial fevers where nervous excitement exists without much pain, in preference to an opiate in any form. Where this one symptom of nervous irritability is uppermost, gelsemium will combine admirably with any antipyretic mixture that may be chosen.

It is claimed by a southern physician of large experience in its use that it is almost a specific in bilious and gastric fevers of children, when pushed till they complain of vertigo and double vision; diaphoresis soon follows, and the little patients are convalescents.

In mania with great motor excitement this remedy is more useful than any of its synergists, in large doses, more even than opium.

In order to obtain the physiological effects of this drug, it must be rapidly introduced, and the moment the system is under its influence its effects upon the eye become as apparent as when atropine or morphia have been taken largely.

The physicians of the South, where it is more extensively used than elsewhere, prefer the strong tincture of the green root, eight ounces to the pint of alcohol.

A tincture or fluid extract made from anything else but the fresh green root is utterly worthless, as the active principle evaporates quickly, even in spontaneous drying.—Dr. HOBB, *in London Med. Journal.*

**On Glycerin in Flatulence, Acidity, and Pyrosis.**—Sydney Ringer, M.D., and William Murrell, report in the Lancet:

An old gentleman, who for many years suffered from distressing acidity, read in a daily paper that glycerin added to milk prevents its souring, and he reasoned thus: "If glycerin prevents milk turning sour, why should it not prevent me turning sour?" And he resolved to try the efficacy of glycerin for his acidity. The success of his experiment was complete, and whenever tormented by his old malady he cures himself by a recourse to glycerin. Indeed he can now take articles of food from which he was previously compelled to abstain, provided always that he takes a dram of glycerin immediately before, with, or directly after his food. He recommended this treatment to many of his friends (sufferers like himself) and one of these mentioned the above circumstances to us.

We have since largely employed glycerin, and find it not only very useful in acidity, but also in flatulence and pyrosis, and that it sometimes relieves pain. We meet with cases where flatulence, or acidity, or pyrosis is the only symptom, but more frequently these symptoms are combined. Some patients rift up huge quantities of wind without any other symptoms than depression of spirits; in others we get flatulence and acidity, one or the other predominating; and we meet with others who suffer from acidity, flatulence and also pyrosis. In all these various forms we find glycerin useful,, and in the great majority of cases very useful. We do not mean to say that in all cases it is superior to other remedies for these complaints; indeed in several instances it has only partially succeeded, where the commonly-used remedies at once cured. On the other hand, in some cases glycerin speedily and completely succeeded, where the commonly-used remedies for acidity and flatulence completely failed. We do not pretend to estimate its relative value to other remedies; we are only anxious to draw attention to its virtues.

Gas is in some instances formed in the stomach, in others in the large intestine, in some patients in both. Our observations were made on stomach flatulence, and as glycerin is so readily absorbed we should hardly expect that it would influence the formation of wind in the colon, except given in large doses, and when it acts as a slight laxative, and so expels the putrefying mass which forms the wind.

In some cases it removes pain and vomiting, probably like charcoal, by preventing the formation of acrid acids, which irritate delicate and irritable stomachs.

We suggest that it acts by retarding or preventing some forms of fermentation and of putrefaction. J. Mekulics (*Archiv. f. Klin. Chir.*) shows that glycerin prevents putrefaction of nitrogenous substances, as of blood diluted with water, which speedily decomposes at the ordinary temperature of the air. Two per cent. of glycerin retarded decomposition for twenty-four hours; ten per cent. for five days. If the fluid were placed in the hatching-oven, then two per cent. retarded decomposition for several hours, ten per cent. for forty-eight hours, and twenty per cent. altogether prevented putrefaction. He also proves that glycerin destroys bacteria and prevents the formation of septic poison, though it will dissolve and preserve the septic poison itself.

Dr. E. Murk (*Virchow's Archiv.*) finds that two to three per cent. will delay lactic fermentation in milk from eighteen to twenty-four hours.

Burnham Wilmot, 1860, says glycerin preserves meat so that after several months' immersion the meat is sweet and can be eaten; and Demarquay proves that both animal and vegetable substances may be kept for from six weeks to two months by glycerin.

Glycerin, however, does not prevent the digestive action of pepsin and hydrochloric acid; hence, while it prevents the formation of wind and acidity, probably by checking fermentation, it in no way hinders digestion. We administer a dram to two drams either before, with, or immediately after food. It may be given in water, coffee, tea, or lemon and soda-water. In tea and coffee it may replace sugar, a substance which greatly favors flatulence, as indeed does tea in many cases. In some instances a cure does not occur till the lapse of ten days or a fortnight.—*Louisville Med. News.*

**Rapid Breathing as a Pain-Obtunder in Minor Surgery, Obstetrics, the General Practice of Medicine, and of Dentistry.**—What led to the discovery of this effect of rapid breathing was in an operation upon myself in 1855, while inhaling chloroform. I was conscious of touch and not pain. I applied it to dentistry in obtunding sensitive dentine, and finally, in 1875, applied it, by increasing the inhalations to one hundred a minute, to the extraction of teeth, and, soon after, to minor surgery, etc. I can in this way render patients insensible to acute pain from any operation where the time consumed is not over twenty to thirty seconds. While the special senses are in partial action, the sense of pain is obtunded, and, in many cases, completely annulled, consciousness and general sensibility being preserved. To accomplish this each patient must be instructed how to act and what to expect. Simple as it may seem, there is a proper and consistent plan to enable you to reach full success.

Before the patient begins to inhale he is informed of the fact that, while he will be unconscious of pain, he will know full or partially well every touch upon the person; that the inhalations must be rigorously kept up during the whole operation without for an instant stopping; that the more energetically and steadily he breathes, the more perfect will be the effect; and if he ceases breathing during the operation the success will not be so complete.

It is very difficult for a person to respire more than one hundred times to the minute, on account of the exhaustion produced. For the next minute following the completion of the operation the subject will not breathe more than once or twice. Very few have force enough left to raise hand or foot. The voluntary muscles have nearly all been subjugated and overcome by the undue effort at forced inhalation of one hundred over twenty—the normal standard. It will be more fully understood, further on in my argument, why I force patients, and am constantly speaking to them to go on.

I further claim that for the past four years, so satisfactory has been the result of this system, in the extracting of teeth and deadening extremely sensitive dentine, that there is no longer any necessity for chloroform, ether or nitrous oxide in the dental office, for such purposes.

The anæsthetics, when used in major operations, where time is needed for the operation, can be made more effective by a lesser quantity when given in conjunction with rapid breathing. Drs. Garretson and Hewson, who have thus tried it, tell me it takes one-half to three-fourths less; and the after-effects are far less nauseating and unpleasant.

As an agent in labor where an anæsthetic is indicated, it is claimed by one who has employed it (Dr. Hewson) in nearly every case for three years, that he has used rapid breathing solely and to the exclusion of chloroform and ether.

If, in breathing, the quantity of carbonic acid gas set free is in exact relation to the amount of oxygen taken into the blood, what effect must be manifested where one hundred respirations in one minute are made while the heart is propelling the blood only a very little faster through the lungs, and more feeble—say ninety pulsations at most—when to be in proportion it should be four hundred to one hundred respirations to sustain any length of time?

You cannot deny the fact that a definite amount of oxygen can be absorbed, as fast as it is carried into the lungs, even if there be one hundred respirations to the minute while the pulsations of the heart are only ninety. Nature has made it possible to breathe so rapidly to meet any emergency, and we can well see its beautiful application in the normal action of both the heart and the lungs while one is violently running.

You are already aware how small a quantity of carbonic acid in excess in the air will seriously affect life. Even two to three per cent. will in a short time prove fatal. In ordinary respirations of twenty to the minute, the average of carbonic acid exhaled is 4.35.

From experiments long ago made by Vierodt (see Carpenter, p. 324) you will see the relative per centage of carbonic acid exhaled from a given number of respirations. When he was breathing six times per minute, 5.5 per cent. of the exhaled air was carbonic acid; twelve times, 4.2 per cent.; twenty-four times, 3.3 per cent.; forty-eight times, 3.0 per cent.; ninety-six times, 2.6 per cent.

Let us deduct the minimum amount—2.6 per cent. of carbonic acid when breathing ninety-six per minute—from the average, at twenty per minute, or the normal standard, which is recorded in Carpenter (p. 524) as 4.35 per minute, and we have retained in the circulation nearly two per cent. of carbonic acid; that, at the average, would have passed off through the lungs without any obstruction, and life equalized; but not having been thrown off as fast as it should have been, it must of necessity be left to prey upon the brain and nerve-centres; and as two to three per cent., we are told, will so poison the blood, life is imperiled, and that speedily.

I think we are now prepared to show clearly the causes which effect the phenomena in rapid breathing.

The first thing enlisted is the diversion of the will-force in the act of forced respiration at a moment when the heart and lungs have been in normal reciprocal action (twenty respirations to eighty pulsations), which act could not be made and carried up to one hundred respirations per minute without such concentrated effort that ordinary pain could make no impression upon the brain while this abstraction is kept up.

Second.—There is a specific effect resulting from enforced respiration of one hundred to the minute, due to the excess of carbonic acid gas set free from the tissues, generated by this enforced normal act of throwing into the lungs five times the normal amount of oxygen in one minute.

Third.—Hyperæmia is the last in this chain of effects, which is due to the excessive amount of air passing into the lungs, preventing but little more than the normal quantity of blood from passing from the heart into the arterial circulation but damming it up in the brain.—*Med. Times*.

**Against the Fever.**—The prophylaxis against yellow fever proposed by Dr. Jos. T. Scotts, of New Orleans, in 1867, and tested by that gentleman and others during many epidemics since is as follows: In the first place, a moderate life, avoiding excesses in eating, drinking and dissipation, but by no means running into etotalism or seclusion. Before breakfast a dose of quinine sulphate, three grains, with lemonade; after dinner three drops of Fowler's solution of arsenic; and twice a day, at convenient intervals, ten-grain doses of chlorate of potash. These for an adult; proper gradations for children.

It will be seen that Dr. Scott's plan includes tne exhibition of three of the most powerful *bacteria-cides* (for want of a better name), each of which has been used in numberless instances as a prophylaxis against yellow fever. One of the chief merits of Dr. Scott's system, however, consists in the alternation or sequence of the remedies, by which means their exhibition can be kept up for a prolonged period without exciting repugnance in the patient and without damage to its digestive powers. Upon the contrary, the effect is immensely tonic. Dr. S. recommends that at the first appearance of the fever all the unacclimated persons, or persons who may fear an attack, should be put upon the regimen indicated, and be kept upon it until the period of general safety has arrived. He has mentioned instances where it has been continued for four months or more. The results, he declares, are that in a great number of instances, unacclimated persons have passed safely through an epidemic closely alongside of such who, without the safeguard, have taken the disease; and that in cases where, in spite of the prophylaxis, yellow fever has been contracted, they have been invariably of a mild and controllable type.

Dr. Scott has the greatest confidence in the system he has proposed; and although he exhibits the natural enthusiasm of the originator, he is a careful physician, of great practical experience in the matters of which he speaks, and his counsel is entitled to great weight. Besides this, his method has had the endorsement of the medical press, and, what is of more worth, of many practitioners in fields where yellow fever commits its ravages.

It seems that the plan is rational, and that if there be anything in prophylaxis, we would put our faith in such as this.—*London Med. News.*

**Chloride of Calcium for Phthisis.**—Dr. Jas. Sawyer, in British Medical Journal, says:

I suppose we are all agreed that cod liver oil, given alone, or variously combined with other agents which tend to promote its assimilation, as with ether, as suggested by Dr. Foster, stands at the head of remedies calculated to promote the general nutrition of the phthisical. Have we any other general remedy? For a long time I trusted to syrup of the iodide of iron. This I gave up for a mixture of hypophosphites and iron—five grains of hypophosphite of lime, ten grains of hypophosphite of soda, and fifteen minims of syrup of the phosphate of iron, for a dose. This is a good combination and I still use it. But chloride of calcium is my favorite drug. I have used it for some years in hospital and private practice, and I believe with great advantage. Perhaps you will say: Do you give it alone? I do not. I give it with cod liver oil, or with cod liver oil emulsion, or with morphia, or with ergot; but my general impression is, *quantum valeat*, that I get better results with chloride of calcium with these combinations than I do with anything else in the same combinations.

My attention was called to the value of chloride of calcium in phthisis by a paper in one of our medical journals, wherein it was stated that the drug was much used by the late Dr. Warburton Begbie. Scarcely mentioned, if noticed at all, in books on drugs, chloride of calcium has an old repute for the cure of strumous glandular swellings. In phthisis I give ten grains, dissolved in a drachm of water and mixed with a drachm of glycerine, in a wineglass full of milk, twice daily, immediately after meals. I think it tends to check night-sweats, to cause increase of weight, and to dry up pulmonary lesions. Of course I do not maintain it does these things in all cases. What I have stated are general conclusions, open to objections, but conclusions which are for me grounds of therapeutic conduct. In prescribing chloride of calcium, we must be careful to write the name of the drug distinctly and in full, in order to avoid an error from which one of my patients suffered, namely, the substitution of "chloride of lime."

**Iodide of Ethyl in Dyspnœa.**—Dr. Robert M. Lawrence, of Boston, in an article on this subject, reported in New York Medical Record, June 10, 1880, submits the following cases :

*Case* 1. Katharine N——, aged fifty, short and of slender build, unmarried, tailoress, first came under the writer's care at the Boston Dispensary, in October, 1876. She had been a martyr to asthma and chronic bronchitis for twelve years. Frequent paroxysms of dyspnœa had greatly reduced her strength, and her sufferings were unusually severe. During the next two years, trial was made of nearly every known remedy, but without much benefit. Tonics and alteratives seemed of no avail.

The nitrite of amyl gave some relief, but was dreaded by the patient on account of its disagreeable physiological effects. In February, 1879, trial was made of ethyl iodide. The result was remarkable. Not only was the dyspnœa relieved, but there was no recurrence of it for several hours, and a good night's rest was obtained. Similar favorable results have followed each inhalation. At the present time, May, 1880, the attacks of dyspnœa are few and far between, and much less severe than formerly.

*Case* 2. James B——, aged fifty-six, slender built indoor man, contracted spasmodic asthma in the army in 1865, and has been subject to it ever since. He had attacks of dyspnœa frequently in the early morning. Has tried most of the usual remedies. In February, 1879, began inhaling ethyl iodide, and found that it gave positive relief. When used at the commencement of a paroxysm, it had the effect of rendering the latter abortive. A decided amelioration of symptoms followed its continued use.

*Case* 3. Thomas A., aged fifty-seven, plasterer, has had nervous asthma for sixteen years. It first supervened on an attack of bronchitis. Paroxysms of dyspnœa were frequent, and lasted some hours. Was obliged to sit up at night. After trial of different remedies, began inhaling ethyl iodide February 14, 1879. Marked relief followed. After several weeks of this treatment, the paroxysms, which had steadily diminished in number, at length ceased altogether.

April 16, 1880. Patient has been free from dyspnœa for a year past, though his respiration is still wheezy.

*Case* 4. Mary M——, aged forty-five years, married, dark complexioned, rather stout, contracted asthma in the following manner: Sixteen years ago, while engaged in frying salt pork, the patient, through some mishap, was nearly suffocated by smoke. An attack of asthma ensued, and she has since been subject to that affection. Paroxysms, on an average, three times weekly. Had tried the ordinary remedies.

April 14, 1880. Half a drachm of the drug was put into a little vial and the patient inhaled the vapor. After three minutes the breathing was easier, and that condition lasted for three hours.

May 4. Has continued to practice three daily inhalations of ten minutes, and had no attack last week.

May 22. She states that the medicine has given more relief than any previous treatment.

*Case* 5. Elizabeth P——, married, aged forty-four years, of nervous temperament, has been subject to a difficulty in breathing for eight years past, which is much aggravated by frequent attacks of bronchitis.

May 19, 1880. After 5 minutes inhalation of ethyl iodide, there was marked exhilaration of spirits and some hysterical laughter. This phase was shortly succeeded by a feeling of invigoration and greater ease in breathing. This condition, together with a sensation of calmness and well-being, lasted for some hours.

May 24. The same effects have followed systematic daily inhalations.

**Toxical Effects of Tea.**—Dr. W. J. Morton, of New York, arrives at the following conclusions concerning the pernicious effects of immoderate tea-drinking:

1. With tea, as with any potent drug, there is a proper and improper dose.

2. In moderation, tea is a mental and bodily stimulant of a most agreeable nature, followed by no harmful reaction. It produces contentment of mind, allays hunger and bodily weariness, and increases the incentive and the capacity for work.

3. Taken immoderately, it leads to a very serious group of symptoms, such as headache, vertigo, heat and flushing of the body, ringing in the ears, mental dullness and confusion, tremulousness, nervousness, sleeplessness, apprehension of evil, exhaustion of mind and body, with disinclination to mental and physical exertion, increased and irregular action of the heart, and increased respiration.

Each of the above symptoms is prodnced by tea-taking in immoderate quantities, irrespective of dyspepsia or hypochondria, or hyperæmia. The prolonged use of tea produces additional symptoms of these three latter diseases. In short, in immoderate doses, tea has a most injurious effect upon the nervous system.

4. Immoderate tea-drinking, continued for considerable time, with great certainty produces dyspepsia.

5. The immediate mental symptoms produced by tea are not to be attributed to dyspepsia. In experimenting upon himself, the whole group of symptoms was produced, with no sign of digestive trouble superadded.

6. Tea retards the waste or retrograde metamorphosis of tissue, and thereby diminishes the demand for food. It also diminishes the amount of urine secreted.

7. Many of the symptoms of immoderate tea-drinking are such as may occur without suspicion of tea being their cause: and we find many people taking tea to relieve the symptoms which its abuse is producing.—*Journ. of Nerv. and Mental Diseases.*

**Eucalyptus Globulus.**—I have used this in the place of a specific for diphtheria—positively saving lives with it. If used early in the disease, it will prevent the membrane from forming in the fauces; and even after it has formed, but before any destruction of tissue, it will remove it quietly and gradually, so as to leave no disfigurement whatever in the parts. If the fever is high, give large doses of it. I have given a quadruple dose, repeating every forty minutes for a few times in a case where the fever momentarily threatened the extinction of the patient, thereby causing him within three hours, to emerge cool and calm, not only from the fever but from the disease itself. I associate this remedy with chlorate potass—alternately, using the latter as a gargle. I sprinkle the eucalyptus also on the meat applied to the throat externally.

It is an efficient remedy, while it is much more merciful to the nerves than quinine is. I will mention a peculiar sequel of the treatment in one case, and leave the reader to judge of the probable cause. There was a swelling of the glands of the throat—slight on the right but immense on the left, the fever being very high at the time. I had just commenced the treatment by eucalyptus, but the doses were ineffectual on account, as I afterward found, of their being much too small (the ordinary doses). I applied to the swelling on the left side, the "druid's hot hand," a peculiar but powerful counter-irritant and anodyne, but did not suppose it necessary to apply the same to the right side. It did its work, while the heroic in eucalyptus, immediately following, soon aborted the disease. An extremely desperate case was quickly cured. Soon a salivation on the right side established itself and continued seven weeks, while all the time the patient (?) was apparently enjoying splendid health. At first the discharge was thick to the feeling of the mouth, and profuse, then became thin and limpid. I determined to do nothing for it, and watched it with some curiosity. Now it is all gone.—*Ther. Gazette.*

**On Tonga.**—The results obtained from Tonga by Drs. Ringer and Murrell fully coincide with mine. I have notes of cases of brain and kidney disease in which tonga alone succeeded in removing pain. I shall, however, confine myself to reporting the effects upon the eye. Some months ago, when commencing experiments with tonga, I had the notion—the result of conversation with patients from the Fiji Islands—that the drug might have a specific effect upon nerves which are instrumental in pain.

Of the three preparations, tonga in a bag, the watery extract, and the alcoholic extract, I found the alcoholic extract alone reliable. When dropped into a healthy eye it seems to increase the power of accommodation, to approach the nearest point of distinct vision, without affecting the size of the pupil (though in some cases, taken in large dose internally, it caused great dilatation of both pupils). It acted beneficially in several cases of asthenopia. The sister in the eye wards gave it with great benefit to a man suffering from painful rheumatic iritis. Several patients with intolerance of light were rapidly relieved.

A most striking effect was obtained upon diminished tension of the eyeball. Two months ago a lady consulted me for intense pain in the right eyeball, with marked decrease of tension (T—2), intolerance of light, and watering, the pupil and cornea being clear, with some conjunctival redness. The intense pain had deprived her of sleep for several nights. Some of the alcoholic extract of tonga was dropped into the eye at 2, 5, 7 and 9 p.m. The following day all intolerance of light had ceased, and she had passed a good night, free from pain. She stated that the drops caused no pain, but a sense of warmth, and that the pain in the eye subsided gradually; their use was continued for several days. Remarkable was the rapidity with which the tension of the eyeball became normal and remained so.

All cases of neuralgia (supra and infra-orbital branches of the fifth nerve), with swelling of the temporal veins during the attack, were benefited. In these a teaspoonful of the extract in half a tumbler of water, and two or three more at an interval of half an hour until the pain subsided, were given.—Dr. Bader, in *Lancet.*

**Diabetes and Sepsis.**—When an individual, apparently in good health, suffers from a progressive gangrenous or ulcerating lesion, for instance on the extremities, when there seems to be no infecting cause, and when all irrigations with carbolic acid prove useless, it is high time to look for diabetes.

This disease causes, very often, these obscure septic processes, and these require rather regulation of diet, with omission of all hydrocarbons, than disinfection with carbolic acid. Correct diagnosis is, in such cases, a matter of the greatest importance, since many patients might be saved if put on the right diet at a sufficiently early period.

Up to the present time three prejudices have often frustrated the diagnosis.

1. It was considered incredible that an individual apparently in good health could have diabetes. By some it was supposed that this disease always produced great thirst and emaciation, and generally cachexia.

2. It is now the fashion to believe that all gangrenous ulcers are caused by bacteria, and to remove these by carbolic acid without searching for any constitutional cause.

3. Even if the diabetes is discovered it is still questioned whether it is the cause of the gangrene, and whether an advanced diabetes can be cured to such an extent that the septic process will cease.

These prejudices are combated by such cases as the following:

Mr. C. R., aged forty-two years, consulted me in June, 1878, on account of progressive gangrenous phlegmasia of the foot. The gangrene involved four toes, spread along the outer edge of the foot to the instep and along the sole to the scaphoid bone. The plantar fascia and superficial tendons were mostly necrotic. The inflammatory infiltration of the ulcer, the red areola, and the swelling of the whole foot, indicated the continuous progress of an infectious phlegmasia. Experience taught me to look for diabetes. This was found present. I then advised animal diet. Although this was not carried out quite to the letter, a quick and noticeable improvement occurred, the gangrene ceased, granulations appeared, and the necrotic portions exfoliated.

The patient entered my clinic July 4, 1878, and Prof. Kulz, our colleague, so familiar with diabetes, had the kindness to watch the diet and the excretion of sugar. He succeeded in reducing the percentage of sugar, which was at first seven per cent. to a mere trifle. After the lapse of ten weeks it was quite plain that a resection of the metatarsal bone of the great toe and of the head of the second metatarsal bone would be necessary, while the rest of the foot could be saved. This was done, and the wounds healed by first intention, so that the patient was able to leave the clinic November 16. He was ordered to continue the animal diet and has since been well.

I might also report two other cases quite similar to this. I have also seen a number of patients, shortly before death, caused by gangrenous or phlegmonous processes, who might probably have been saved had the diagnosis been made in time.—*Med. Gazette.*

**Wolfe's Operation for Cataract.**—The London Medical Times and Gazette states that Dr. Wolfe's method of obviating the risk of failure in cataract extractions is thus noticed in the current number of the *Centralblatt fur Practische Augenheilkunde.*

In cases of infantile cataract, Dr. Wolfe opens the capsule, and two or three days later he removes the softened lens with a broad needle, rendering, thereby, the use of pumping unnecessary. In senile cataract he makes, two or three weeks before, a narrow iridectomy downward, in such manner as not to interfere with the ciliary border of the iris. For the removal of the lens he uses speculum, forceps, and Graefe's knife, with which he opens 1''' more than the third part of the corneal circumference at its scleral border, leaving a narrow bridge. Speculum, knife and forceps are then put aside, the capsule is opened, the bridge divided with a very small cornea knife, and the cataract removed by soft digital pressure.

The use of chloroform is avoided.

Traumatic cataracts, when *in situ*, are treated in the same manner; when dislocated forward, they are extracted without iridectomy; when luxated backward, they are brought into the anterior chamber and then removed. We recently had an opportunity of witnessing the elegant performance of this operation, and convinced ourselves of the safety of the method.—*Med. and Surg. Reporter.*

**Ovariotomy.**—On Friday, June 11th, Mr. Spencer Wells performed ovariotomy for the one thousandth time. When we remember how many thousand hours of thought and anxiety, in addition to direct surgical labor, this unparalleled feat represents, we may well admire the completion of a Herculean task; yet sober reasoning gives us yet more cause to admire its commencement, both as a credit to surgical science and to the great contemporary operator himself. The thousandth case was the brilliant consummation, under relatively easy conditions, of a struggle commenced under diametrically different auspices.

Let us bear in mind what the word ovariotomy implied when it caught the ear of any surgeon in December, 1857; then let us reflect on the present position of the operation.

In addition to Mr. Wells' long list of cases, we have long tables of statistics of operations by other surgeons, which, but for Mr. Wells' example, might have been, at the least, much shorter. Fairness leaves us room for but one opinion as to whom honor and credit are due.

**What Becomes of Blood and of Different and Indifferent Foreign Bodies in General in the Joints.**—Fresh blood which had been injected into the kneejoint of rabbits became, after a half hour, partly coagulated, and remained partly in its liquid state. After 1½ to 15 hours, the liquid portion had disappeared from the joint; the third part of the blood injected adhered, in a coagulated condition, to the wall, and was already on the third day lined with the endothelium of the sac, and traversed by cellular bands.

Those coagula found loose in the articular cavity were also coated with layers of cellular tissue.

On the tenth to fifteenth days all coagula had disappeared; for several weeks following there was only pigment noticed on the wall of the sac. Indifferent and well disinfected bodies (slate chips, sand, well chopped gauze) introduced into the cavity produced at first sweling of the joint, and became also coated with endothelium.

Particles of iron, however, did not become encysted, and organic substances (well pounded muscular fibres) were absorbed like blood. Completely boiled globules of quicksilver caused suppuration in the joint followed by abscesses spreading into the soft parts.

**Asthma.**—Dr. Sheffer, of Bremen, thinks that in most cases of asthma there will be found, on examination, chronic inflammation of the upper parts of the air passages (pharynx, larynx and trachea). These should always be attended to first. Then the asthma may be cured by the judicious use of an induced current of electricity. The currents should not be very weak, and the electrodes should be placed on each side of the neck.

Dr. S. does not claim that this will effect a cure in all or even in the majority of cases, but he reports sixteen cases thus treated with eight cures. Considering the nature of the disease, these figures are sufficiently encouraging to lead one to try the treatment with electricity, especially as no harm can result. Dr. S. thinks it of especial importance to first treat any inflammation of the pharynx.—*Physician and Sur.*

**A Certain Remedy for Diphtheria.**—The Boston Medical and Surgical Journal, April 8, 1880, states that Henri Bergeron reports that hydrofluoric acid evaporated in the proportion of one gram to each cubic meter in the sick-room, and thus inhaled by the patient, is a certain remedy for diphtheria. Three hours should be consumed in the evaporation.

He says that all who submitted to this operation for forty-eight hours recovered.—*Med. Gazette.*

