# Correction

**Published:** 1880-09-20

**Authors:** 


*Correction.*—The article in our August issue on gelseminum, credited to London Med. Journal, Dr. Hobbs, ought to have been credited to Louisville Med. News, Dr. A. G. Hobbs.

